# Supplementary material for: Assembly of complex viruses exemplified by a halophilic euryarchaeal virus
Source: Nat Commun. 2019 Mar 29;10:1456. doi: 10.1038/s41467-019-09451-z (PMC6441041; doi:10.1038/s41467-019-09451-z)
Supplement: Supplementary file 1 — Supplementry Information [file 41467_2019_9451_MOESM1_ESM.docx]

**Assembly of complex viruses exemplified by a halophilic euryarchaeal virus**

Luigi De Colibus, Elina Roine, Thomas S. Walter, Serban L. Ilca, Xiangxi Wang, Nan Wang, Alan M. Roseman, Dennis Bamford, Juha T. Huiskonen & David I. Stuart

**Supplementary Information**

**Figs. 1-11**

**Tables 1-2**

**Supplementary Figure 1. Effect on cryo-EM maps of correction for imperfect symmetry and focus gradient. The upper image of each colour-matched pair shows the map before and the lower image after correction.**

**
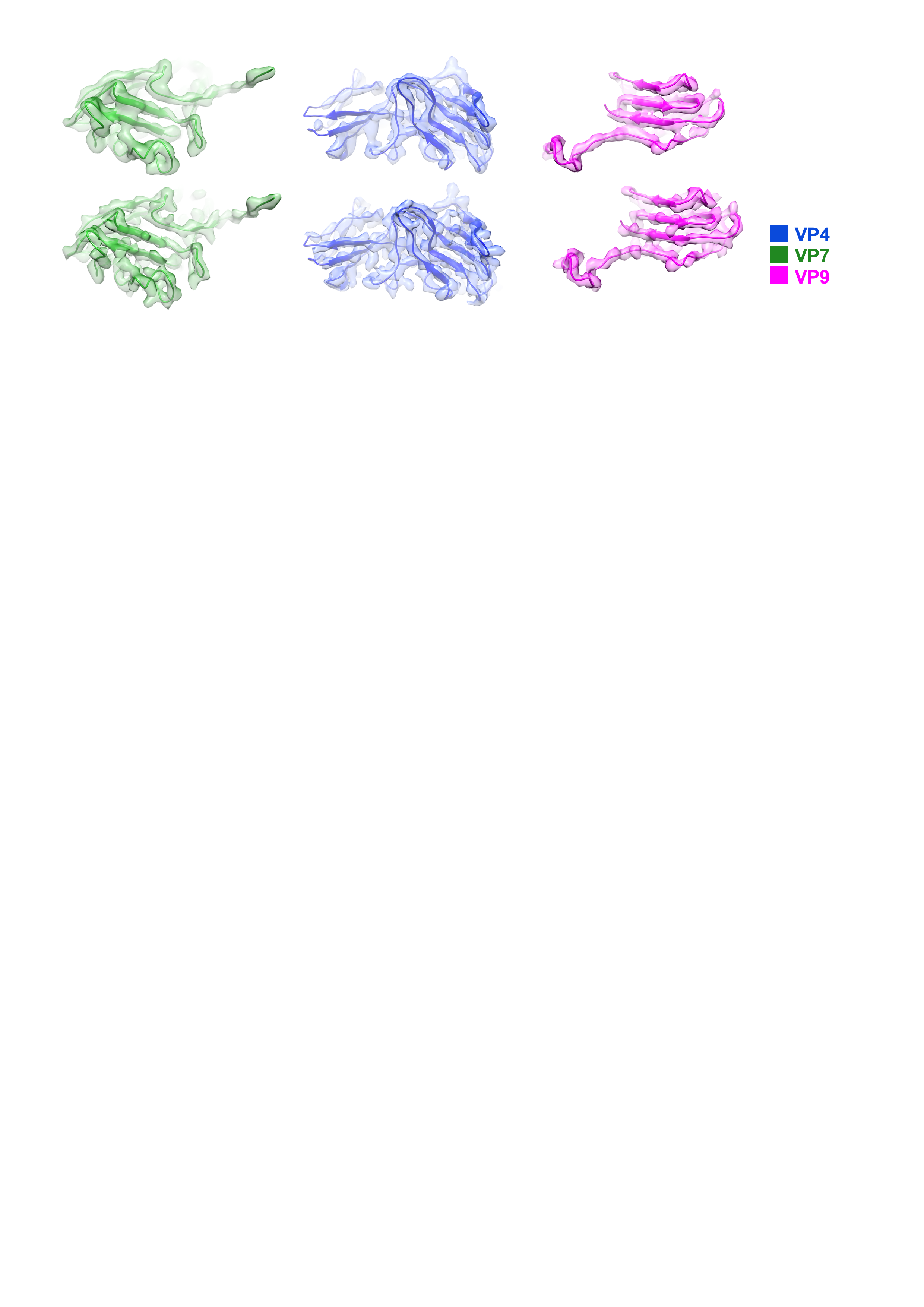
**

**
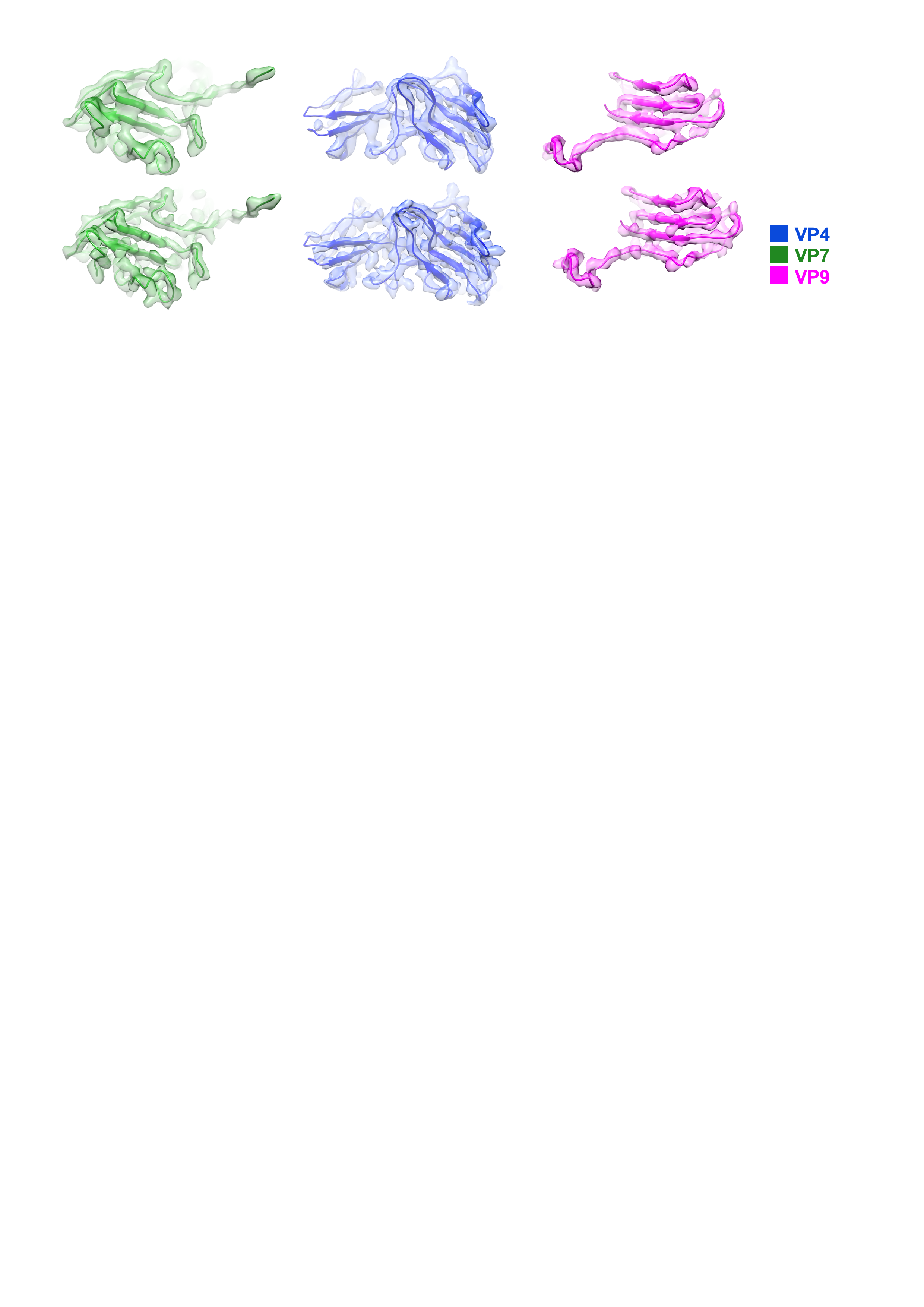
**

**Supplementary Figure 2. Graphs of Fourier shell correlation (FSC) between two independent half sets of data (from Relion, see Methods). (a–c)** Block-based reconstructions of three blocks termed penton (a), vertex-adjacent (b), and two-fold adjacent (c). **(d–f)** Localized reconstructions of the capsid asymmetric unit (d), ‘horn’ with C2 symmetry (e), and without symmetry (f). Volume masking (Masked) was used to exclude the solvent region and this increased the estimated resolution compared to the original unmasked reconstruction (Unmasked). Possible effects of the masking were compensated for by noise randomization (Randomized) to create the final FSC curve (Corrected). The resolution at which the correlation drops below the FSC=0.143 threshold is indicated.

| **(a) 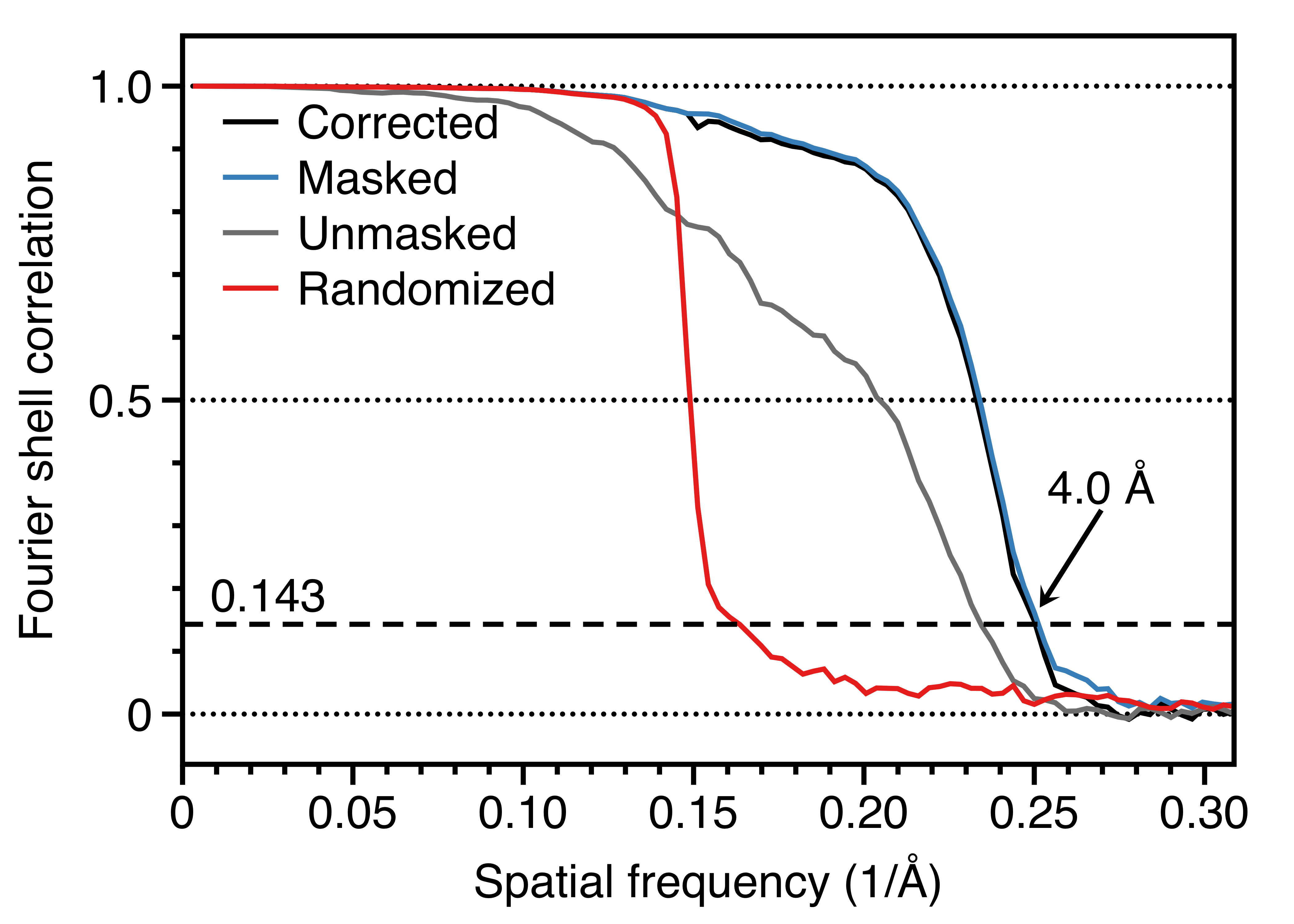** | **(b) 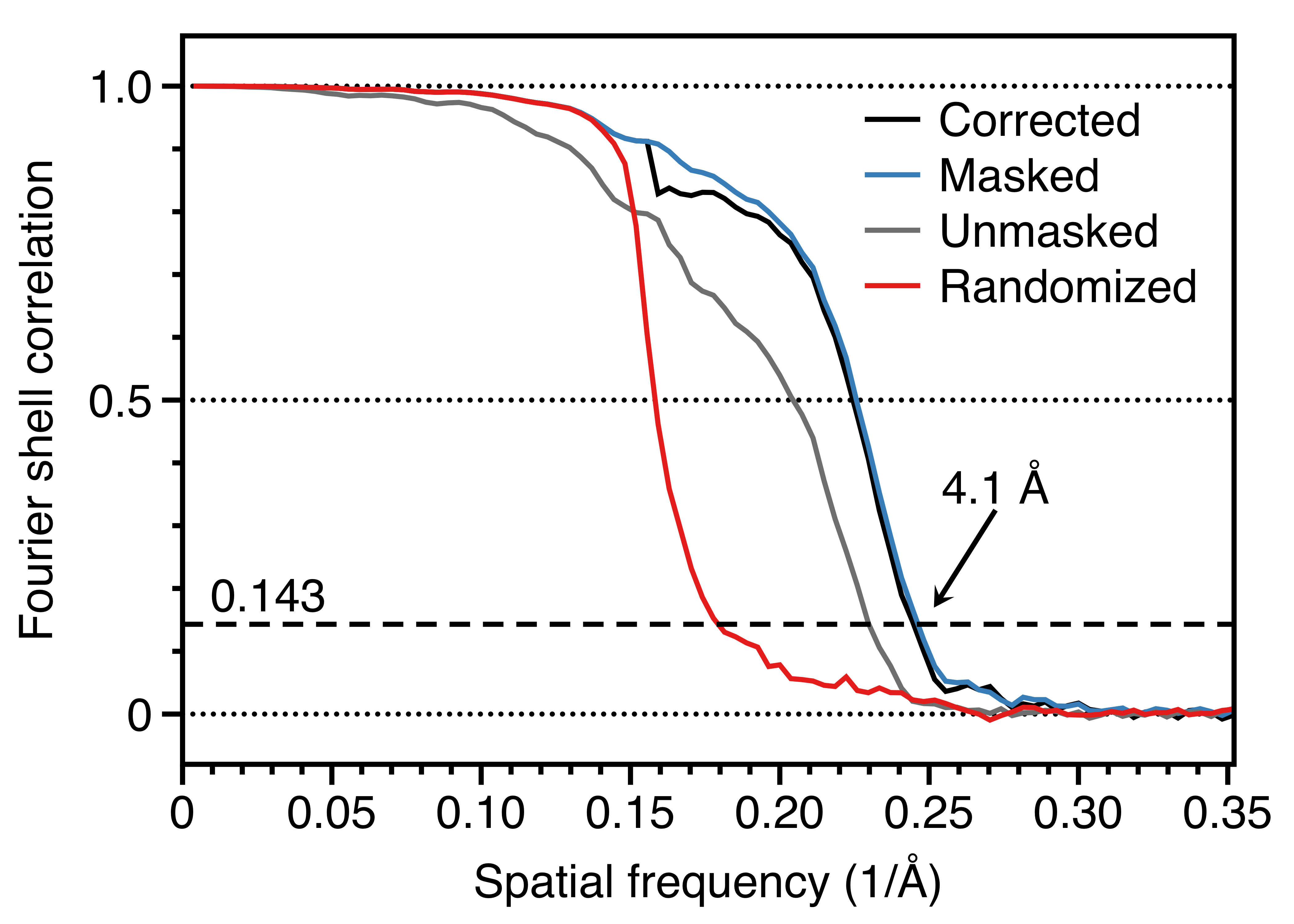** |
| --- | --- |
| **(c)**  **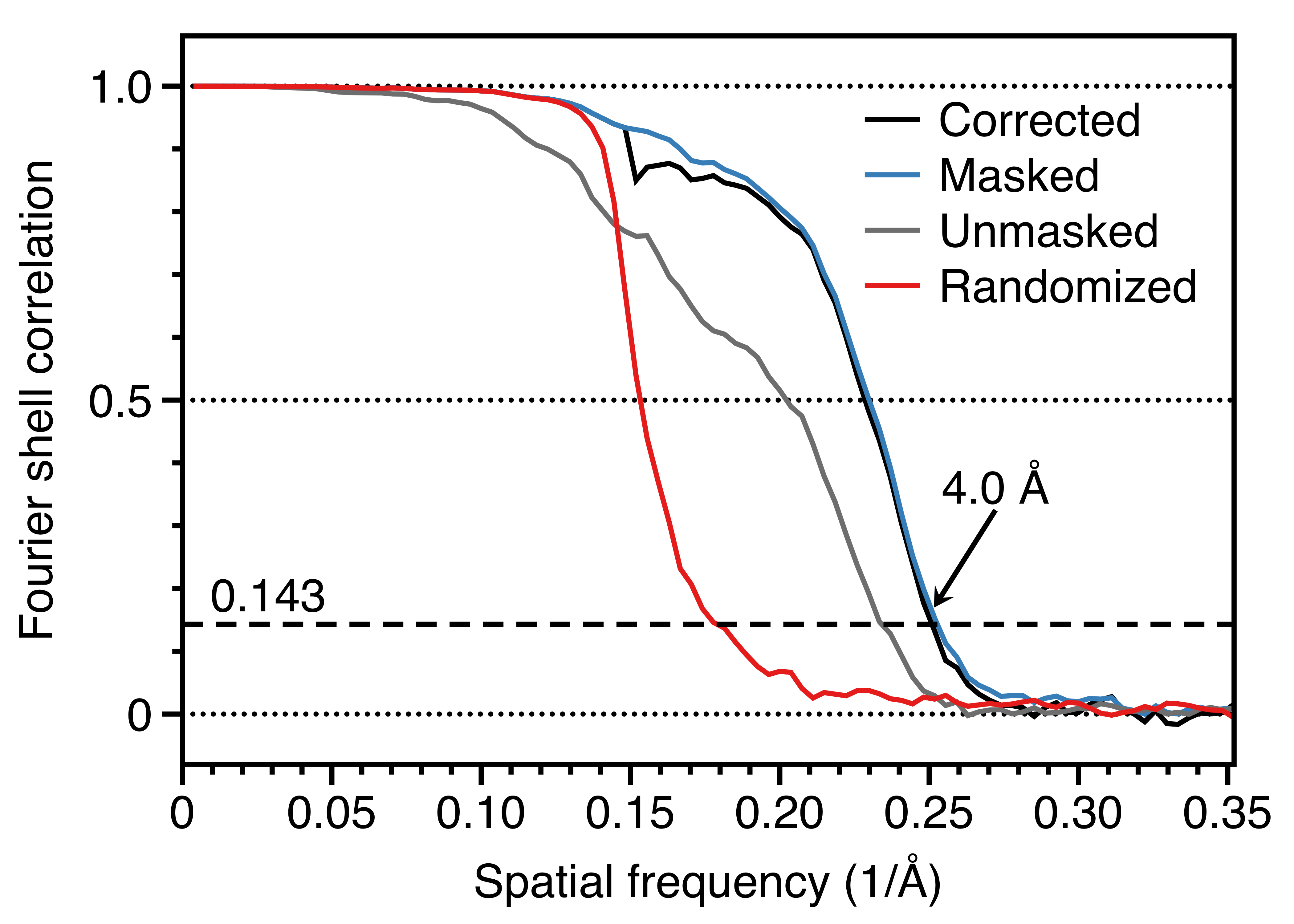** | **(d)**  **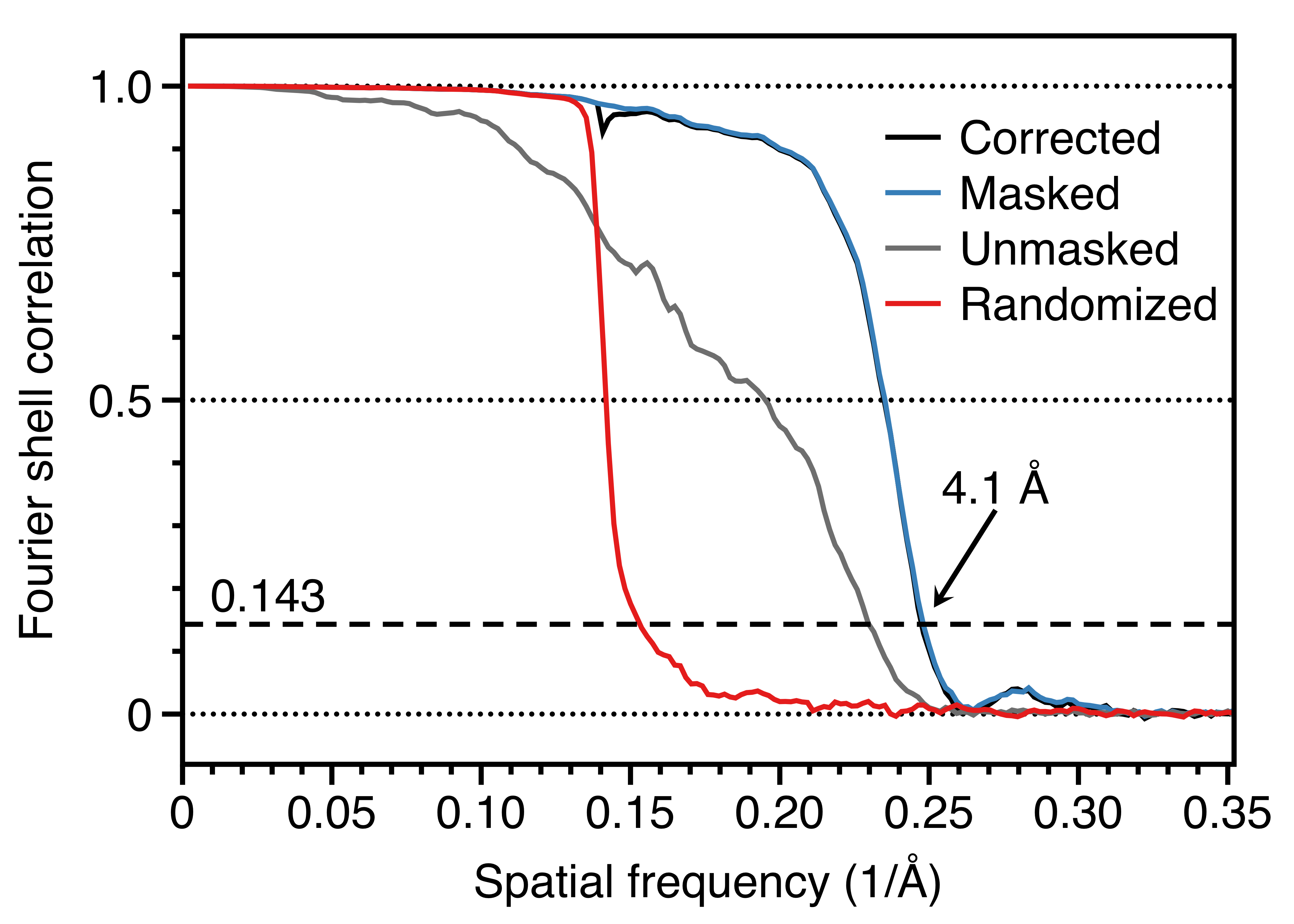** |
| **(e) 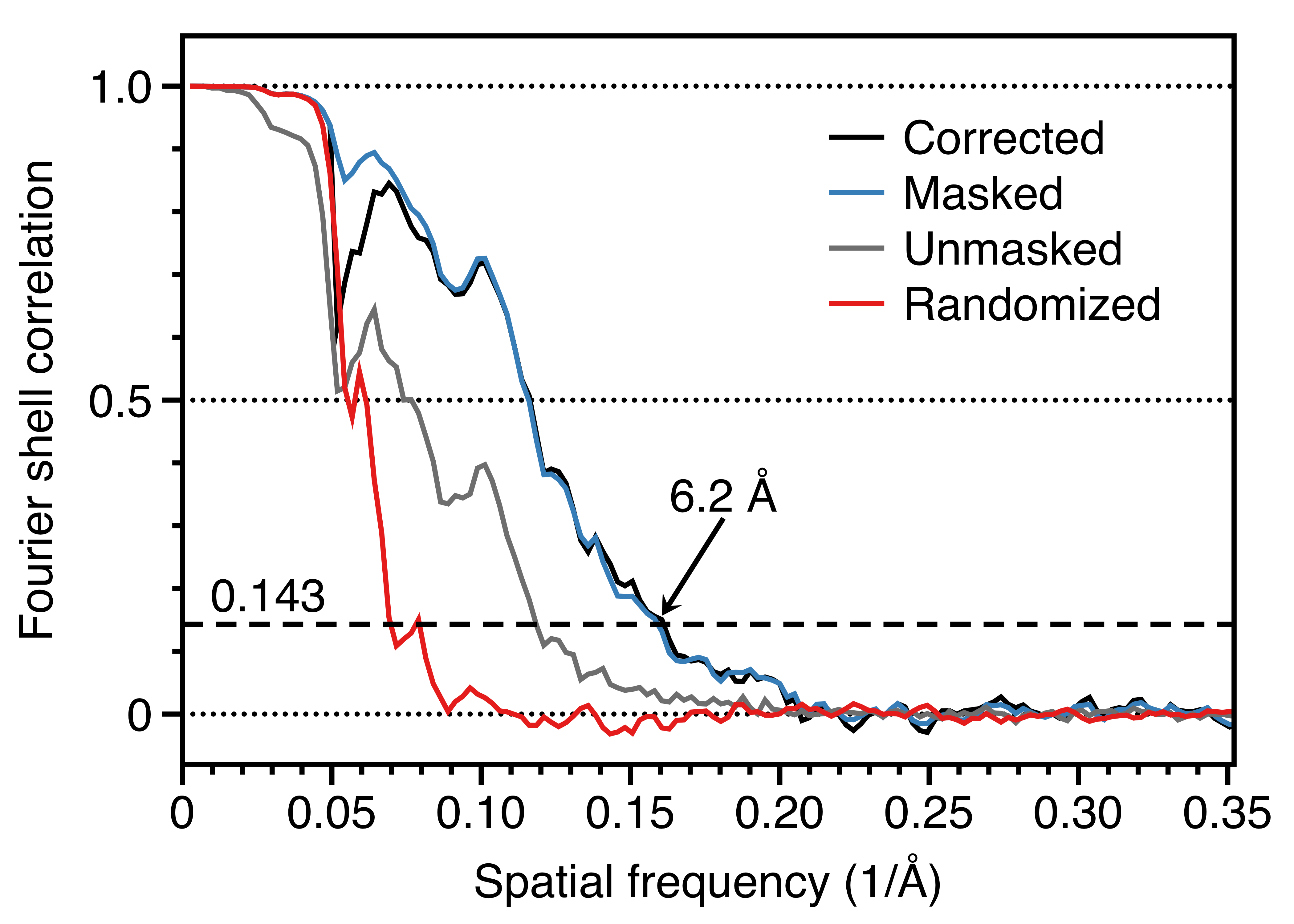** | **(f) 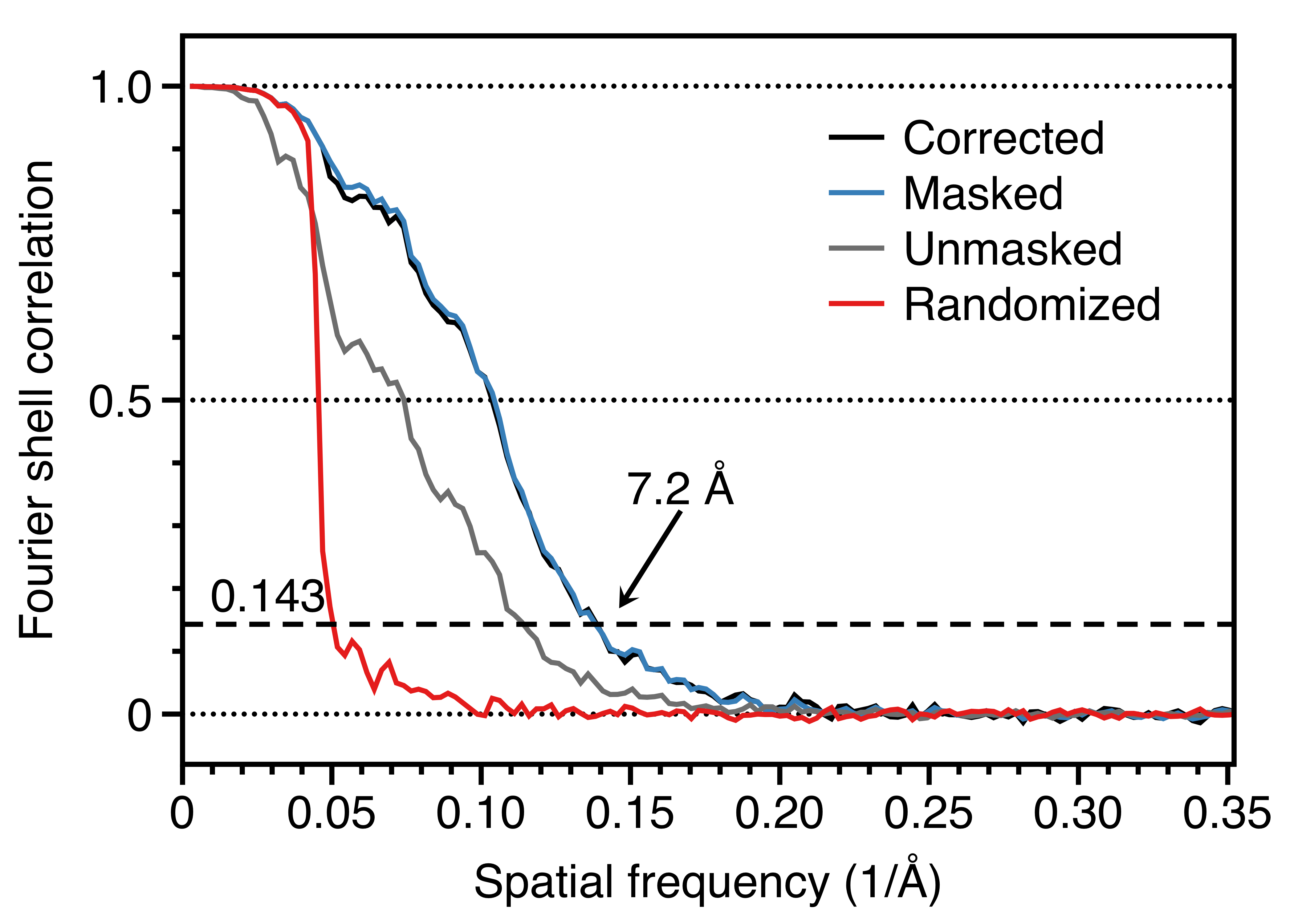** |

**Supplementary Figure 3. Graphs of Fourier shell correlation (FSC) between two half sets of data from block-based refinement (from cisTEM, see Methods).** The map was divided into units (vertex/penton, vertex-adjacent and 2-fold axis adjacent blocks) and then combined into a single region (penton). Particle shows the FSC before correction.


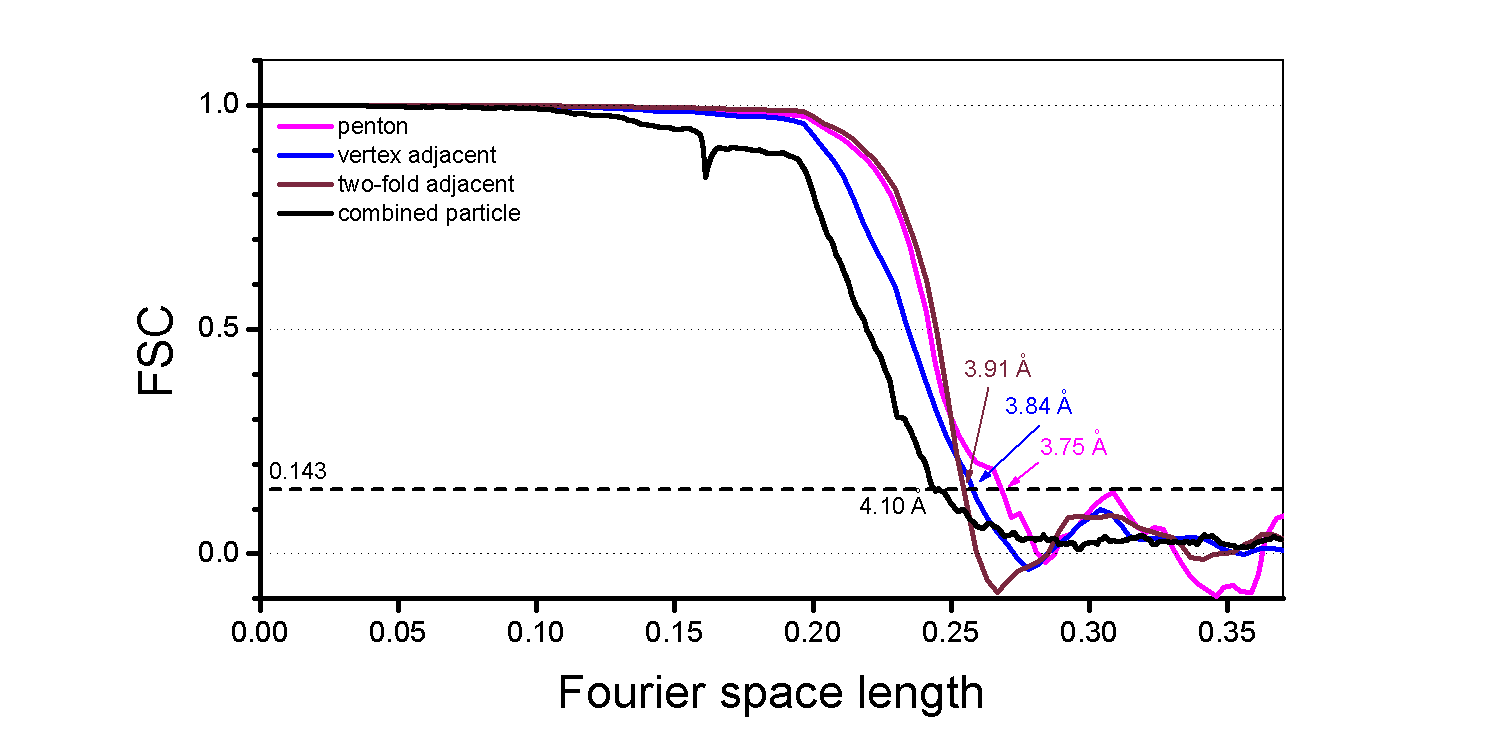


**Supplementary Figure 4. Local resolution analysis of C1 map of the vertex (scale shows resolution in Å) (a) top view, (b) side-view.**

**(a)**

**
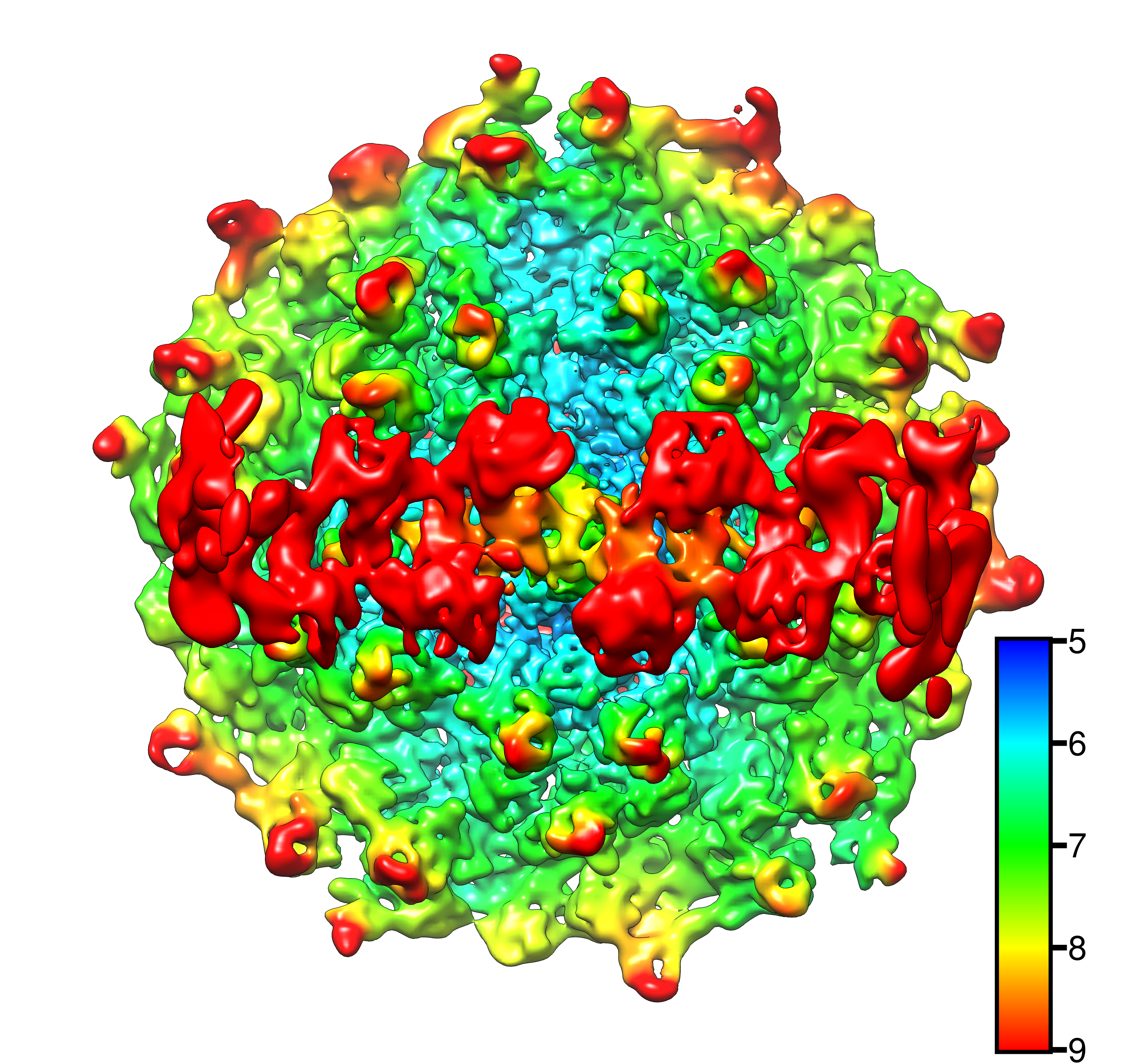
**

**(b)**

**
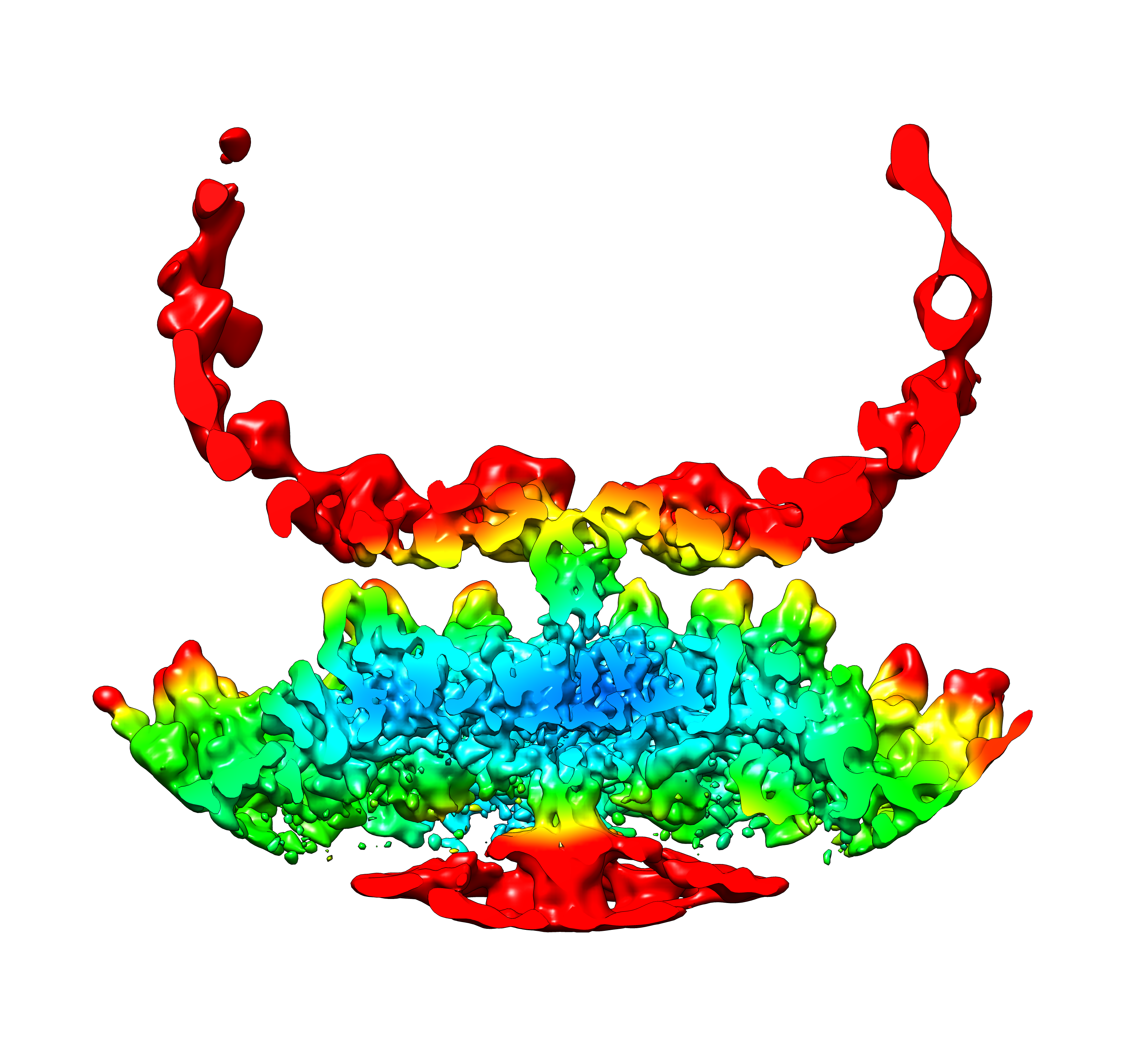
**

**Supplementary Figure 5. Penton protein comparison. VP9 (magenta) of SH1 compared to P31 of PRD1 (gray).**

**
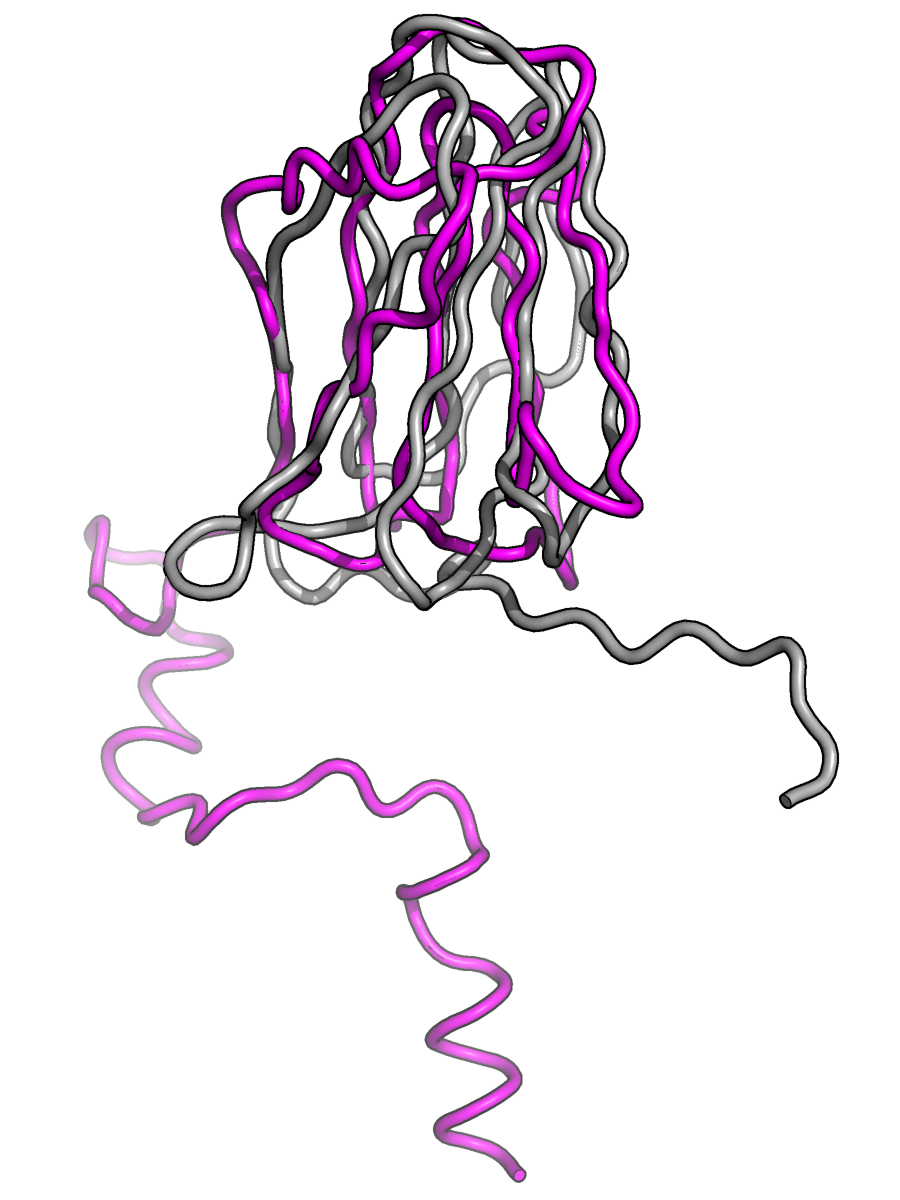
**

**Supplementary Figure 6. Comparison of the MCPs of SH1 and P23-77.**

1. **P23-77 VP17 (orange) on SH1 VP4 (blue).**

**
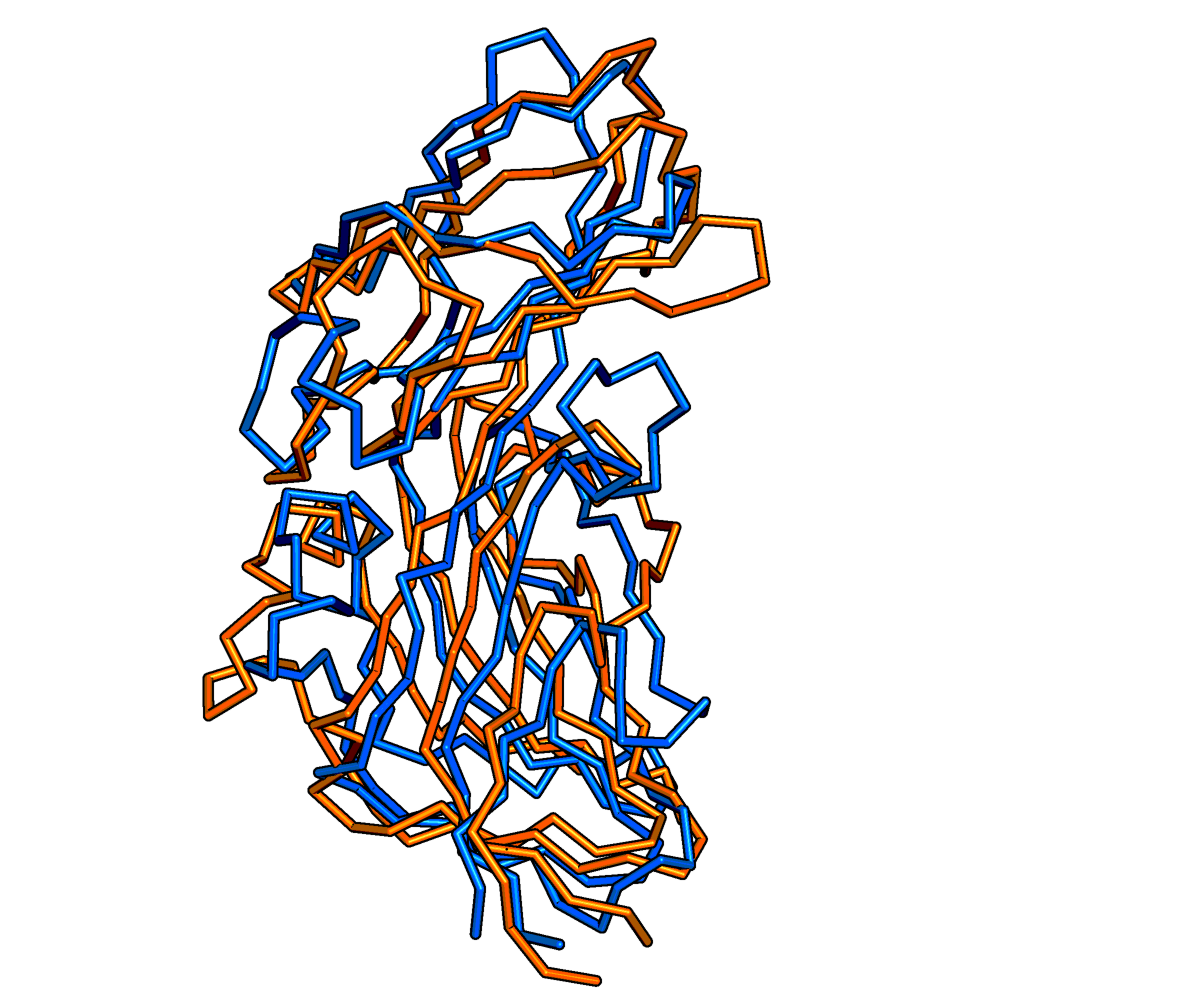
**

1. **P23-77 VP16 (grey) on SH1 VP7 (green).**

**
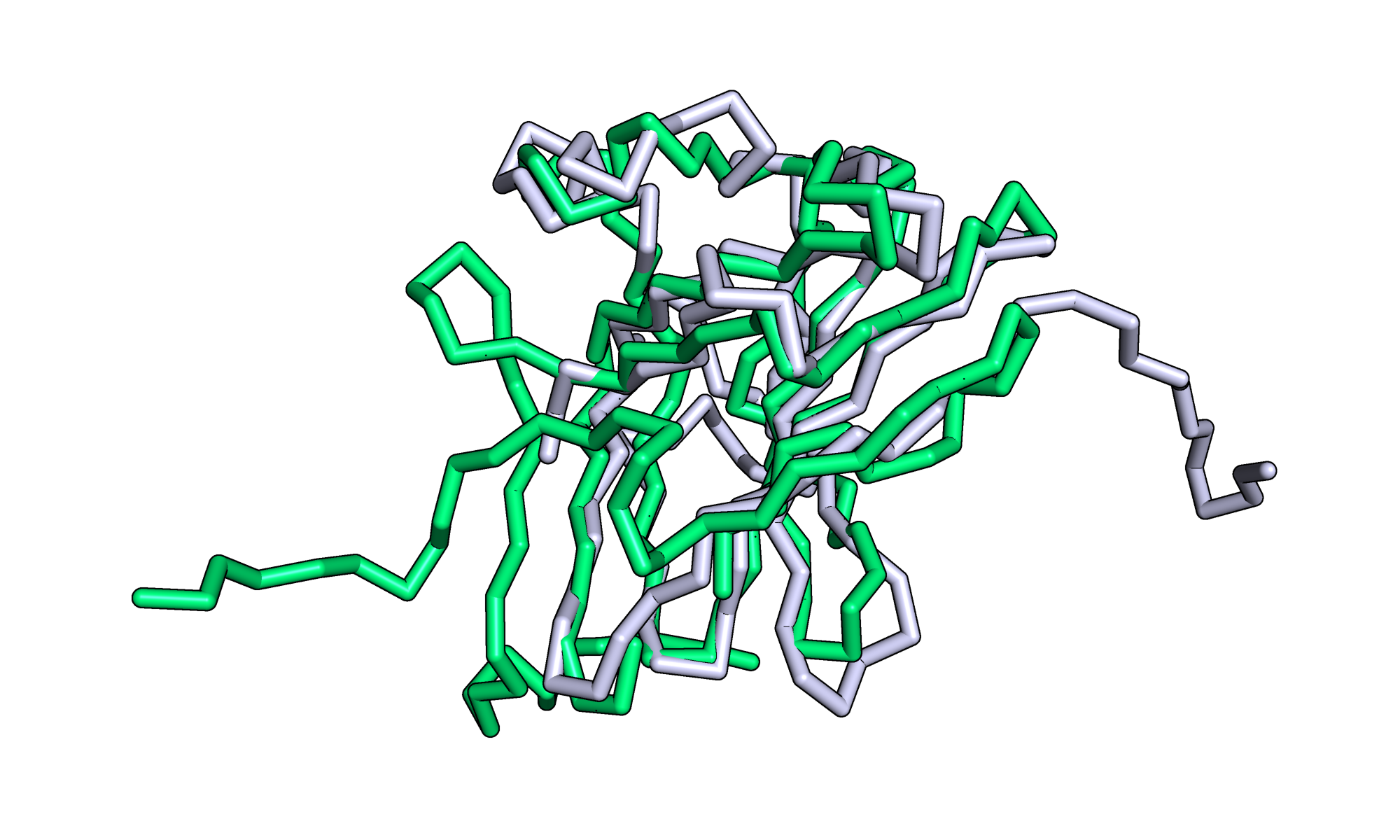
**

**C**

**C**

**Supplementary Figure 7. Comparison of VP7-VP4 heterodimer and PRD1 P3 subunit.**

**VP7 green, VP4 blue, P3 gray.**

**
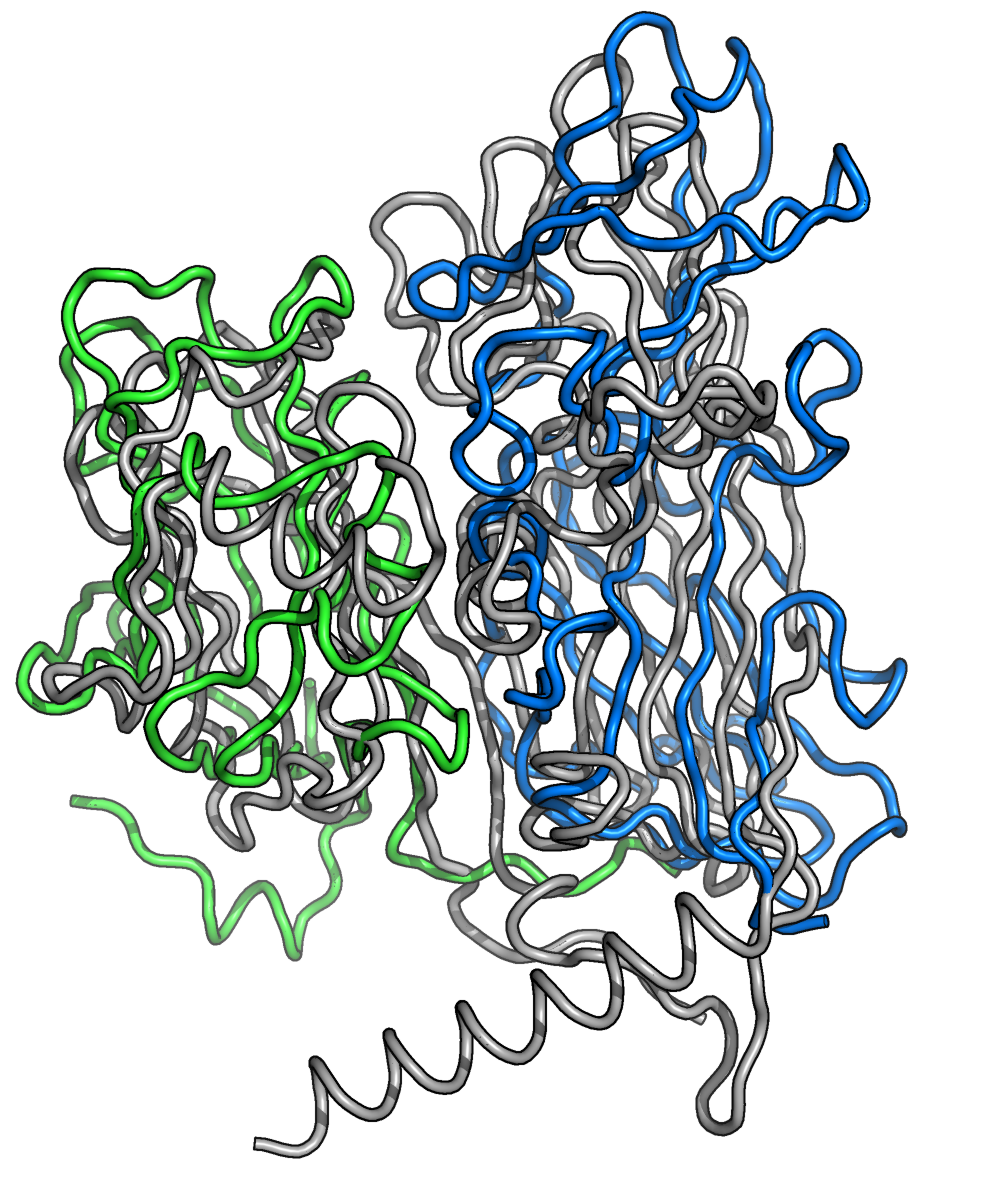
**

**Supplementary Figure 8. PSIPRED (**<http://bioinf.cs.ucl.ac.uk/psipred/>) **server output for protein VP13.**

**Supplementary Figure 9. Comparison of minor protein density below the type III 5-fold adjacent and the type II hexamers.** Each pair of images shows 2-fold averaged density for the minor protein density into which the putative structure of VP13 is fitted. The VP13 structure has also been expanded by 2-fold symmetry. (a) and (b) show side and top views for the density associated with the type-III 5-fold adjacent hexamer, (c-f) show corresponding views for the type II 2-fold adjacent (c) and (d) and type II 2-fold hexamers (e) and (f).


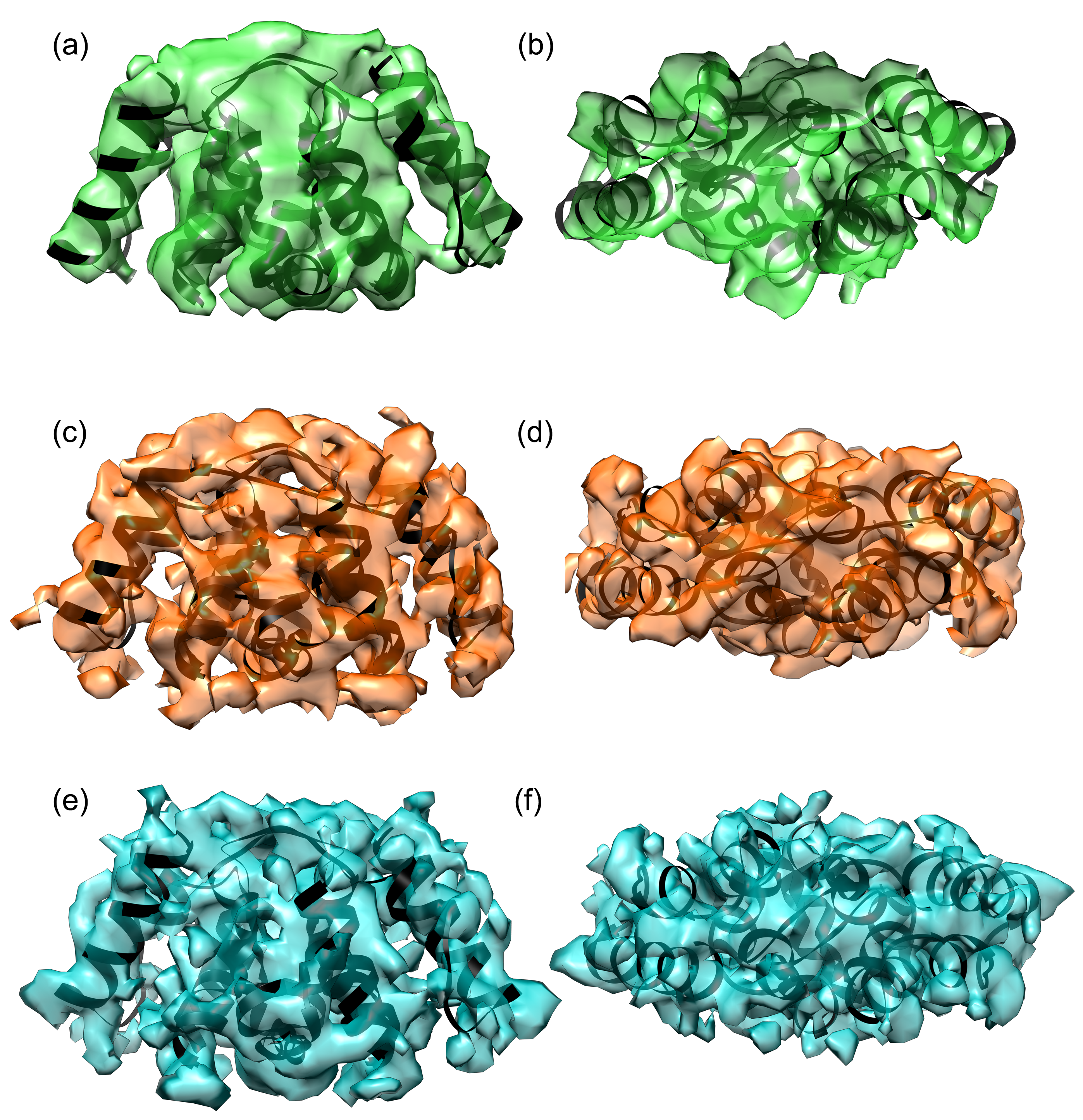


**Supplementary Figure 10. Symmetry mismatch at the icosahedral 5-fold.**

Localized reconstructions of the capsid asymmetric unit (map calculation described in Methods). (a) side view showing the two connections of the spike to the penton (b) top view (taken at the level shown in (a) by a grey line) the connections are marked with yellow stars. Additional density at the clockwise related subunits is marked with green stars.

**(a)**

**
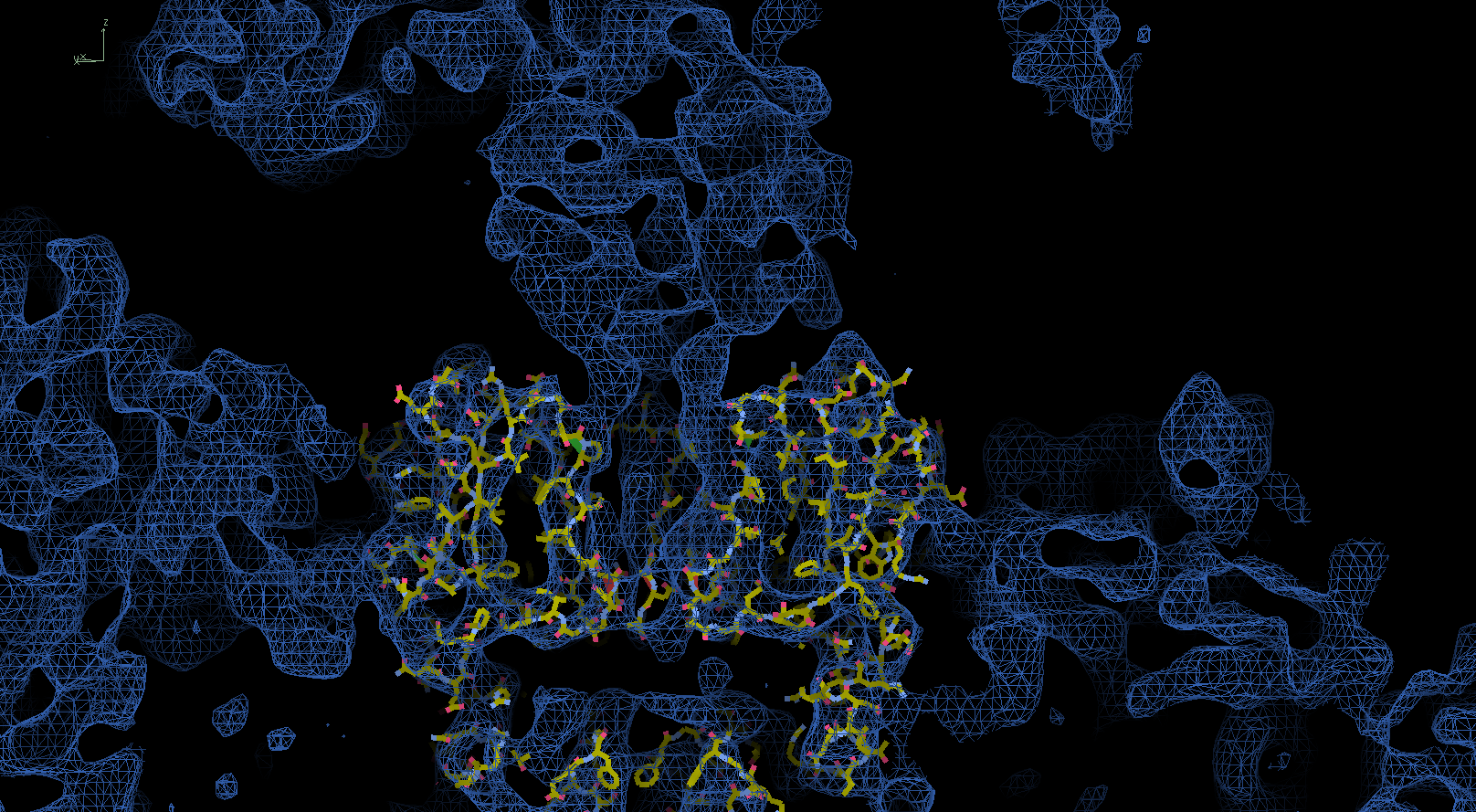
**

**(b)**

**
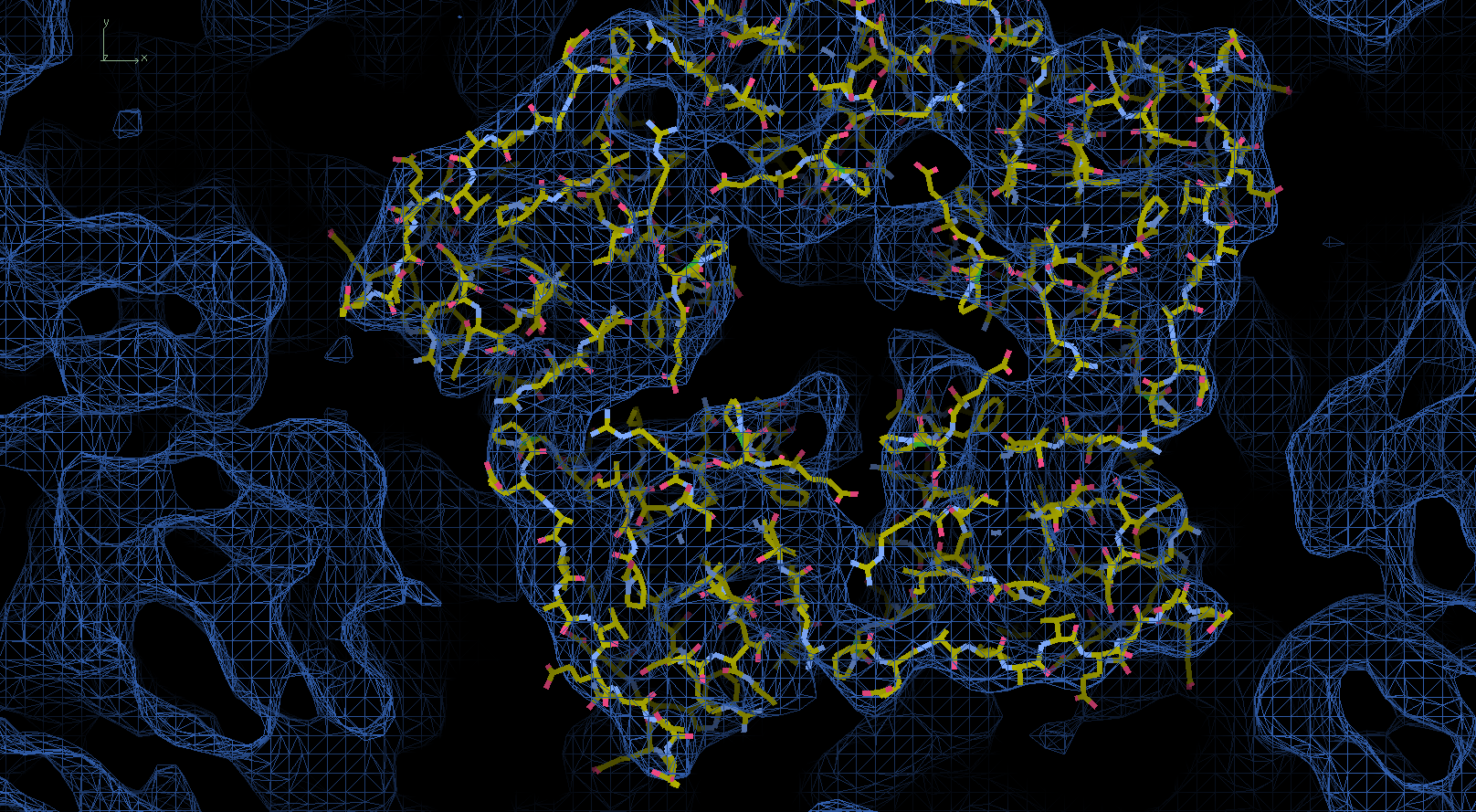
**

*

*

*

*

**Supplementary Figure 11. The effect of averaging and then sharpening the density of the non-icosahedrally related MCPs.** (a-b) VP4 residues 135-142: (a) unaveraged, representative subunit, (b) averaged. (c-d) VP7 residues 124-134: (c) unaveraged, representative subunit, (d) averaged.

**(a)

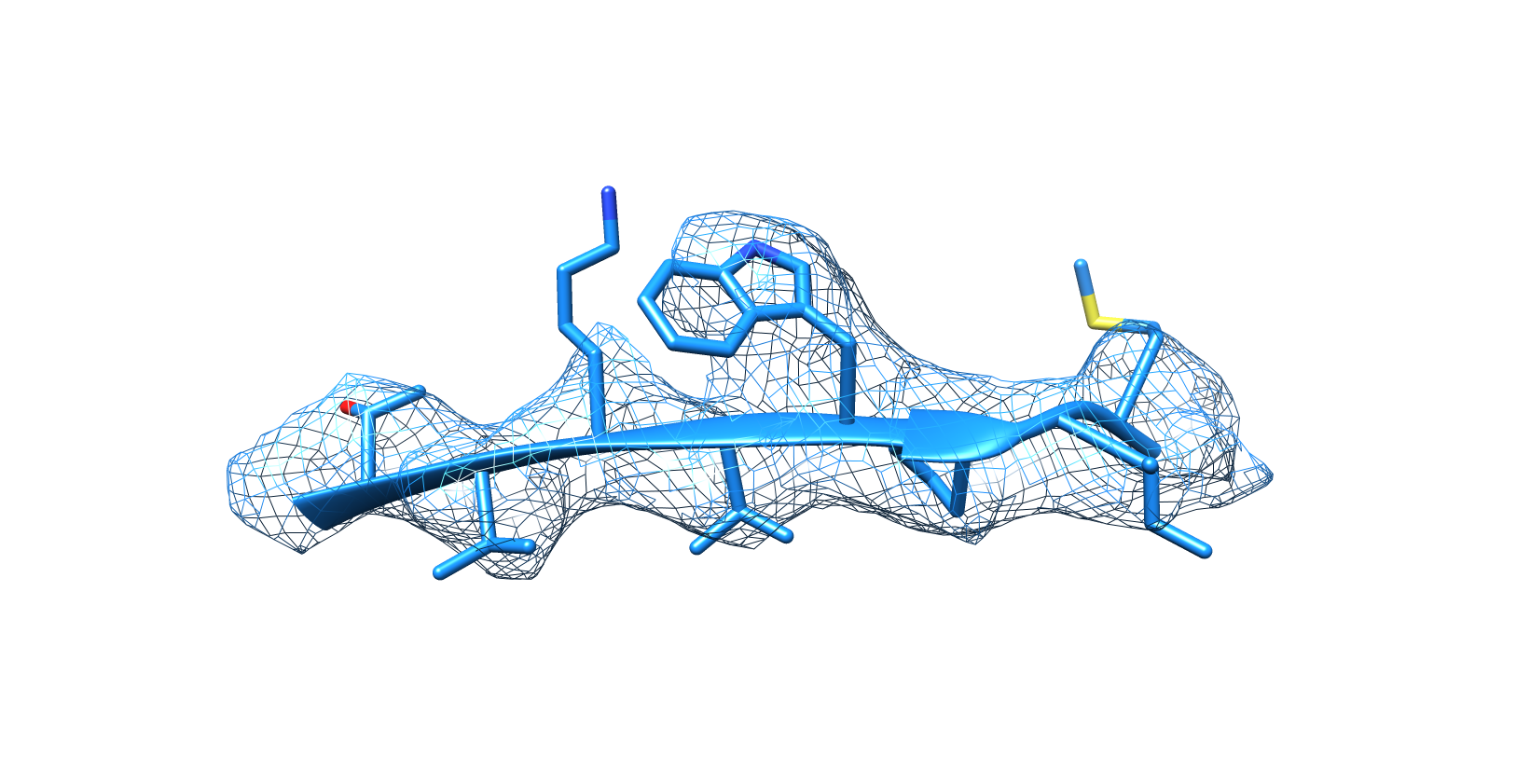
(b)
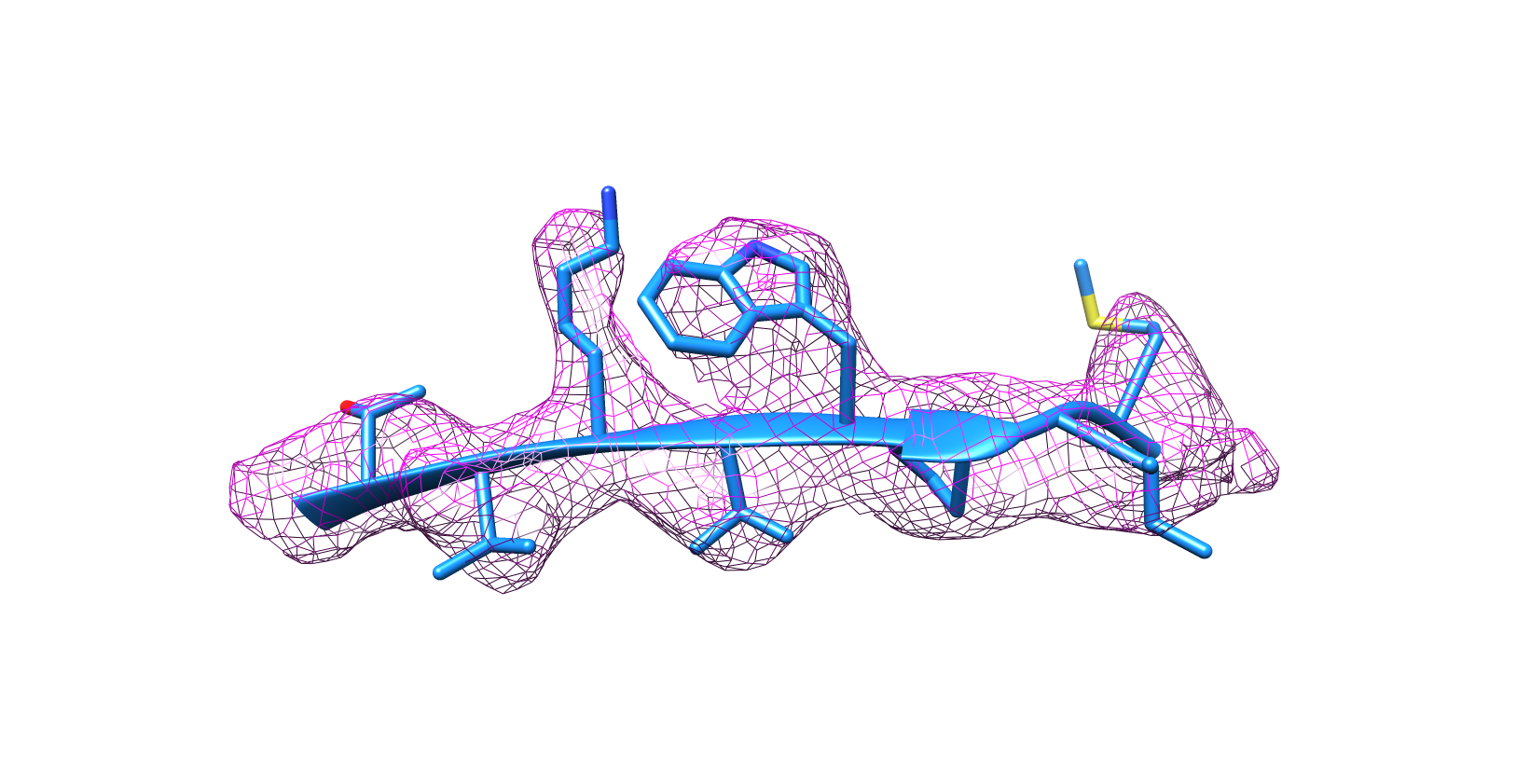
(c)
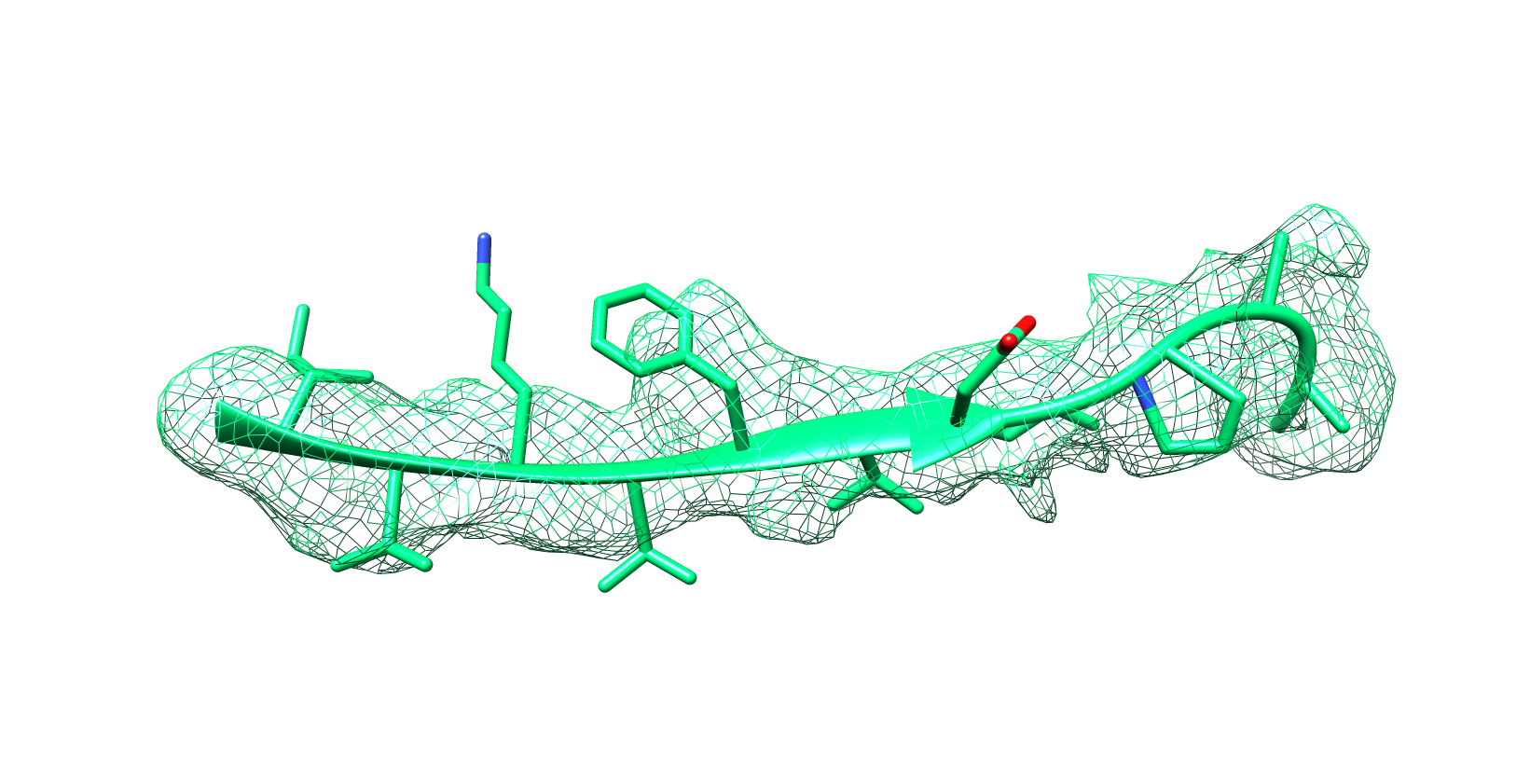
(d)
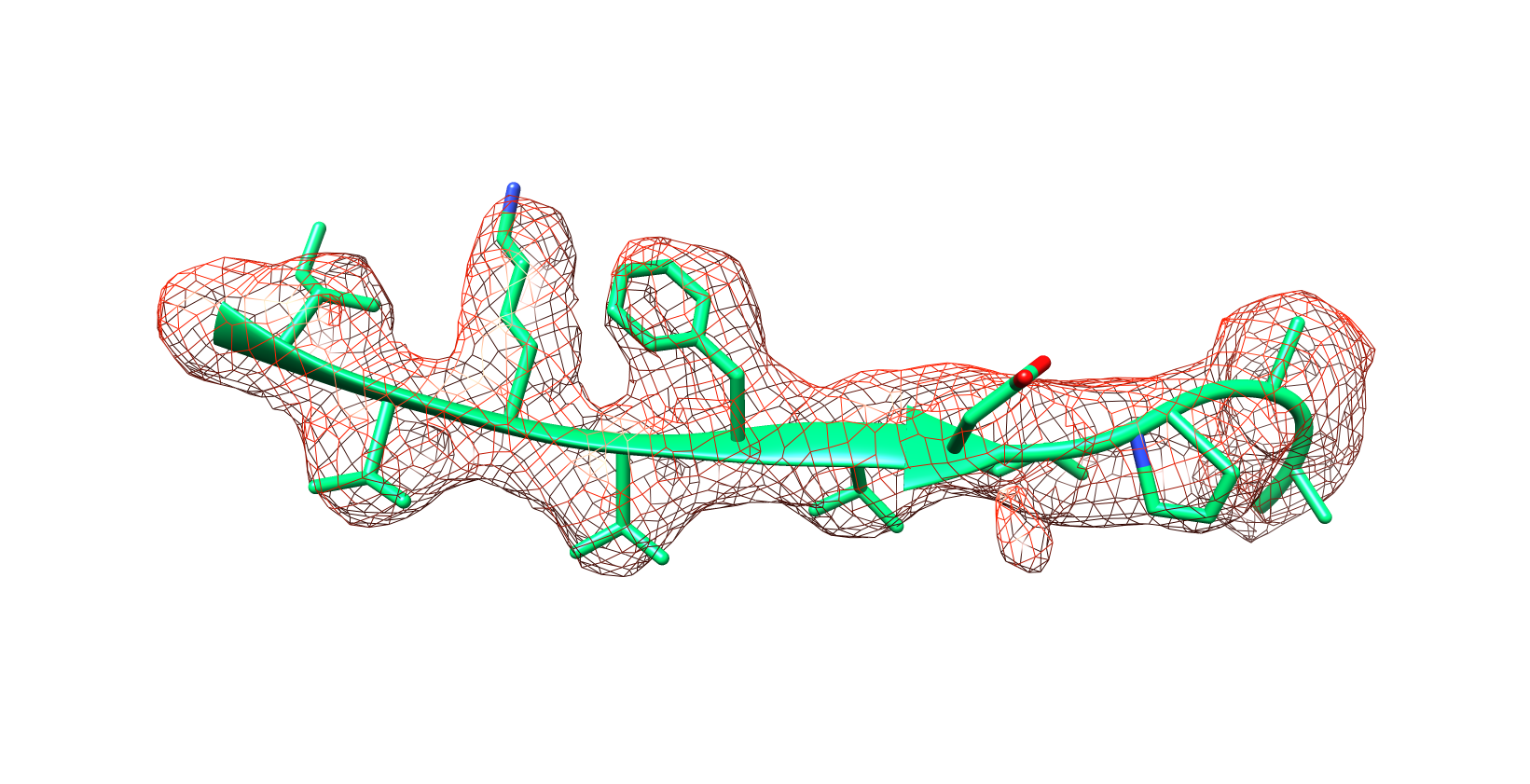
**

**Supplementary Table 1. Cryo-EM statistics

(a) Data collection and processing**

| **Parameter** | **Value** |  |  |  |  | |  |  |
| --- | --- | --- | --- | --- | --- | --- | --- | --- |
| **Cryo-EM data collection** |  |  |  |  |  | |  |  |
|  | Full Particle (Relion 1.4) | Spike | Vertex | Block-based technique | |  |  |  |
| Voltage (kV) | 300 | 300 | 300 | 300 | |  |  |  |
| Magnification (×) | 37,037 | 37,037 | 37,037 | 37,037 | |  |  |  |
| Defocus (μm) | 0.7–2.5 | 0.7–2.5 | 0.7–2.5 | 0.7–2.5 | |  |  |  |
| Dose rate (e^–^/pixels/s) | 8 | 8 | 8 | 8 | |  |  |  |
| Frames | 22 | 22 | 22 | 22 | |  |  |  |
| Frame length (s) | 0.2 | 0.2 | 0.2 | 0.2 | |  |  |  |
| Total dose (e^–^/Å^2^) | 22 | 22 | 22 | 22 | |  |  |  |
| Micrographs | 1775 | 1775 | 1775 | 1,381 | |  |  |  |
| **Cryo-EM data processing** |  |  |  |  | |  |  |  |
| Particles | 3,918 | 9,866 | 11,990 | 16,185 | |  |  |  |
| Box size (pixels) | 800 | 300 | 300 | 800 | |  |  |  |
| Pixel size (Å) | 1.35 | 1.35 | 1.35 | 1.35 | |  |  |  |
| Symmetry | I | C2 | C1 | I | |  |  |  |
| Resolution (Å) | 4.3 | 6.2 | 7.2 | 3.8 | |  |  |  |
| Sharpening B-factor (Å^2^) | -155 | -255 | -248 | -90 | |  |  |  |

**(b) Capsid protein (VP4, VP7, VP9, VP12, VP13) structure refinement and validation for the full capsid.**

|  | |
| --- | --- |
| **Parameter** | **Value** |
| Resolution (Å) | 3.8 |
| Number of atoms | 4923600 |
| CC_mask | 0.73 |
| CC, volume | 0.74 |
| **RMS deviations** |  |
| Bonds (Å) | 0.01 |
| Angles (º) | 1.3 |
| **Average B-factor (**Å^2^) | 144 |
| **Molprobity*** |  |
| Molprobity score, asymmetric unit  Clashscore, asymmetric unit | 1.98  6.8 |
| Ramachandran (% favored) | 87.86 |
| Ramachandran (% allowed) | 11.97 |
| Ramachandran (% outliers)  Rotamer outliers (%)  Cβ deviation (%)  CaBLAM outliers (%)  CA geometry outliers (%) | 0.07  0.02  0.0  4.6  0.9 |
| **EMRinger** score** | 1.24 |

* Chen *et al.* (2010) Acta Crystallographica D66:12-21.

** Barad et al. (2015) Nature Methods 12:943–946.

**Supplementary Table 2. Interaction areas between subunits forming the icosahedral asymmetric unit.** Uppercase subunits (columns ‘Range’) are VP4 and lowercase VP7.

**
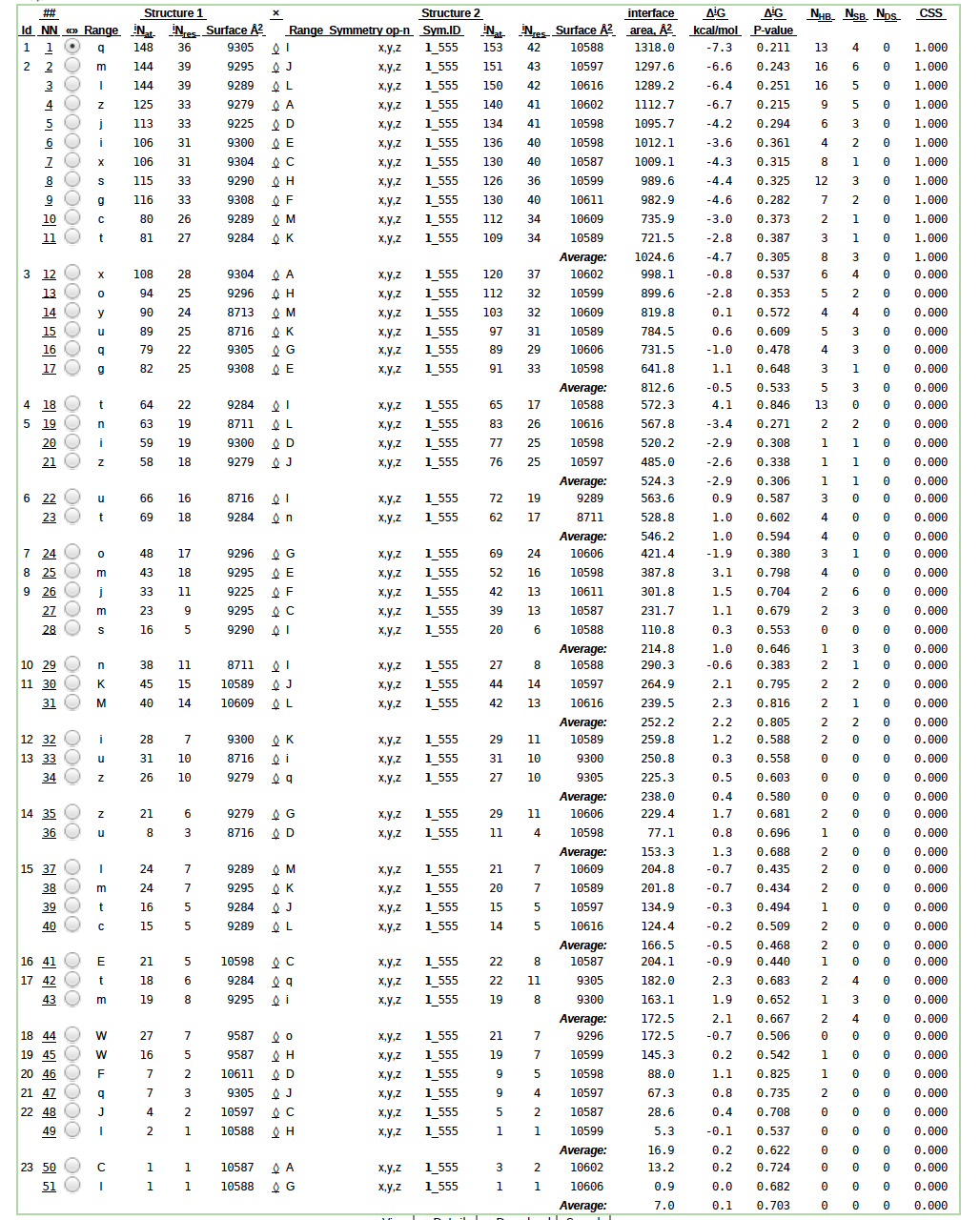
**
